# 

**Published:** 1886-06

**Authors:** 


### OUR ADVERTISERS.

SEE new advertisement of Bellevue Hospital Medical College.

PEACOCK'S BROMIDES.—Drs. Robb & Hall, Woodburn, Kentucky, say: We find Peacock's bromides to be one of the best remedies we have ever used in nervous headaches, and in cases where a nerve sedative is indicated it acts admirably.

R. A. ROBINSON & CO., LOUISVILLE.—We desire to direct attention to the advertisement of this firm in this issue. It is one of the oldest as well as one of the most reliable houses in the United States, and their goods are reliable in every respect.

MASSANETTA WATER may be had, plain or carbonated, of Heister & Co., Memphis; Gooding & Shugrue, Knoxville; J. H. Hill & Co., Goldsboro, North Carolina; Z. A. Barnes, Eufaula, Alabama; J. O. Pattan, Montgomery, Ala.; and Charles O. Tyner, Atlanta, Georgia.

ARE YOU GOING AWAY THIS SUMMER?—Great enthusiasm seems to prevail among the summer resorts along the line of the East Tennessee, Virginia and Georgia Road, the Norfolk and Western and Shenandoah Valley Railroads, and crowds are already booked for the East Tennessee and Virginia mountains. The excursion rates will be lower this year than ever before, and circulars and schedules can be secured upon application to ticket agents throughout the South, or to B. W. Wrenn, general passenger agent, Knoxville, Tennessee.

LACTATED FOOD.—The Wells & Richardson Co., of Burlington, Vermont, manufacturers of this food, have received the following letter from J. Milner Fothergill, M. D., of London:

GENTLEMEN—Having requested me to give you my opinion, as a food expert, upon your "Lactated Food," I do so herewith. You state that it contains "the purified gluten of wheat and oats with barley diastase and malt extract combined with specially prepared milk sugar;" in other words, that it is self-digestive as regards the conversion of insoluble starch into soluble dextrine and maltose. My experiments with it lead me to hold that this is correct.

The food then contains carbo-hydrates, some albuminoid matter and the various salts in grain, notably phosphate of lime.

Such a food can be added to milk and treated in the manner you describe in your leaflet. So prepared with milk it forms an admirable food for infants and dyspeptic persons who require very digestible aliments.

But it has a wider range of utility. The body temperature is kept up by the combustion of grape sugar. Grape sugar is supplied from carbo hydrates, either the insoluble starch or the soluble sugar. Starch forms a great portion of our food and is converted into grape sugar within the body. Where the system is unequal to the digestion of starch, as in feeble digestion, or conditions of acute disease, then predigested starch must be furnished to the organism. Otherwise the system will perish of exhaustion, just as fire dies out when its fuel is consumed.

Beef tea contains nothing which can form grape sugar, and in fact is a pleasant stimulating beverage or food adjunct, but without food value practically. (For what food value it has is so infinitesimal that it is not worth counting.) But when it has added to it a food such as your lactated, food it has a distinct measurable food value. Consequently such food should be given with beef tea, and the compound forms a valuable food.

When lactated food is placed in water hot enough to be sipped, a rapid transformation of the starch remaining in it (by the diastase it contains) goes on, and a nutritive fluid is the result, which requires but a minimum of the digestive act.

Such fluid can be flavored and drank as a nutritive beverage, specially acceptable in febrile conditions. Flavored with lemon, ginger, cloves or other flavoring agents to give variety—a matter far too much neglected in the treatment of the sick—it can be largely used. Or wine, either red wine as claret, or sherry or port, can be added to it when a little stimulant is required, and brandy when a stronger stimulant is indicated.

The resort to farinaceous matters, predigested, must become greater and greater as our knowledge of digestion and its derangements waxes larger. It is not merely in the case of feeble infants that such predigested starch and milk sugar are indicated and useful; persons of feeble digestion require these soluble carbo-hydrates which they can assimilate.

But to my mind an equally great matter is the feeding of persons acutely sick, and especially when there is pyrexia, who now are allowed to perish of inanition on the mistaken conviction that beef tea is a sustaining food. It is in the sick-room that soluble carbo-hydrates have a great future before them.

J. MILNER FOTHERGILL, M. D.

